# A multi-gene phylogeny of *Chlorophyllum* (*Agaricaceae*, *Basidiomycota*): new species, new combination and infrageneric classification

**DOI:** 10.3897/mycokeys.32.23831

**Published:** 2018-03-20

**Authors:** Zai-Wei Ge, Adriaana Jacobs, Else C. Vellinga, Phongeun Sysouphanthong, Retha van der Walt, Carmine Lavorato, Yi-Feng An, Zhu L. Yang

**Affiliations:** 1 Key Laboratory for Plant Diversity and Biogeography of East Asia, Kunming Institute of Botany, Chinese Academy of Sciences, Kunming 650201, China; 2 National Collection of Fungi, Biosystematics Division, ARC, Plant Health and Protection, Queenswood 9012, Pretoria, South Africa; 3 111 Koshland Hall 3102, University of California at Berkeley, Berkeley, California 94720-3102, USA; 4 Ecology Division, Biotechnology and Ecology Institute, Ministry of Science and Technology, P.O.Box: 2279, Vientiane Capital, Lao PDR; 5 C/da Calamia 10 – I-87069 San Demetrio Corone (CS), Italy

**Keywords:** *Agaricales*, *Lepiota*, *Macrolepiota*, multigene phylogeny, new taxa

## Abstract

Taxonomic and phylogenetic studies of *Chlorophyllum* were carried out on the basis of morphological differences and molecular phylogenetic analyses. Based on the phylogeny inferred from the internal transcribed spacer (ITS), the partial large subunit nuclear ribosomal DNA (nrLSU), the second largest subunit of RNA polymerase II (*rpb2*) and translation elongation factor 1-α (*tef1*) sequences, six well-supported clades and 17 phylogenetic species are recognised. Within this phylogenetic framework and considering the diagnostic morphological characters, two new species, *C. africanum* and *C. palaeotropicum*, are described. In addition, a new infrageneric classification of *Chlorophyllum* is proposed, in which the genus is divided into six sections. One new combination is also made. This study provides a robust basis for a more detailed investigation of diversity and biogeography of *Chlorophyllum*.

## Introduction

The genus *Chlorophyllum* Massee, 1898 (*Agaricaceae*, *Agaricales*) is typified by *Chlorophyllum molybdites* (G. Mey.) Massee. This genus currently accommodates ca. 16 species (Kirk et al. 2011) and 30 records can be found in Index Fungorum (http://www.indexfungorum.org/Names/NAMES.ASP). Traditionally, this genus was monotypic, only containing the green-spored species, *C. molybdites*. Based on similarities in morphology and/or molecular evidence, a few species previously placed in *Macrolepiota* Singer or *Lepiota* (Pers.) Gray, were transferred into it (Vellinga 2002). Similarly, *Endoptychum agaricoides* was also transferred to this genus based on molecular evidence and the proposal to conserve *Chlorophyllum* (hereafter abbreviated as *C.*) against *Endoptychum* was submitted to retain the genus name for the well-known toxic species *C. molybdites* (Vellinga and De Kok 2002) and accepted (Gams 2005). Members of this genus are characterised by the following unique combination of morphological characters: the pileus covering is hymenidermal, the stipe (if present) is smooth and basidiospores lack a germ pore or have a germ pore caused by a depression in the episporium without a hyaline covering. The basidiospores are white, green, brownish or brown in deposit and the habit varies from agaricoid, secotioid to gasteroid (Crous et al. 2015a; Ge and Yang 2006; Vellinga 2003a; 2004b; Vellinga et al. 2003). Species within this genus are saprotrophic and distributed worldwide, often growing in urban and ruderal habitats, with a preference for tropical and subtropical regions (Vellinga 2004a).

Recently, three species, *C. lusitanicum* G. Moreno, Mohedano, Manjón, Carlavilla & Altés, *C. pseudoglobosum* J. Sarkar, A.K. Dutta & Acharya and *C. sphaerosporum* Z.W. Ge & Zhu L. Yang were described from Spain, India and China, respectively (Crous et al. 2015a; Crous et al. 2015b; Ge and Yang 2006). These studies provided a better understanding of the species diversity within the genus, but are confined to certain specific regions and samples from other poorly explored areas such as Africa have seldom been included. Such studies have been focused on new species descriptions, but an infrageneric classification for the genus is still lacking because infrageneric relationships are poorly known.

Phylogenetic studies have shown that *Chlorophyllum* is nested within *Agaricaceae* (Ge and Yang 2017; Vellinga 2004b; Vellinga et al. 2003; Vellinga et al. 2011). However, due to limited taxon sampling and /or use of the ribosomal RNA genes only (ITS and /or nrLSU), limited information on infrageneric relationships could be gleaned. Further sampling of more species and phylogenetic analyses based on protein coding genes are needed to clarify relationships within *Chlorophyllum*.

Based on investigations of lepiotoid fungi in China, Dominican Republic, Germany, Italy, South Africa, Thailand, United Kingdom and the United States of America, detailed morphological and molecular studies were carried out in this study. The aims were to:

1. elucidate species diversity within *Chlorophyllum* based on both morphological characters and phylogenetic analysis, describe novel species and provide more information on poorly known species;

2. use a combined multi-gene dataset (ITS, nrLSU, *rpb2* and *tef1*) to provide a robust hypothesis for relationships amongst *Chlorophyllum* species;

3. examine diagnostic characters for recognised clades and establish an infrageneric classification that best reflects the evolutionary history of the genus.

## Materials and methods

### Taxon sampling and morphological studies

Fifty-nine collections were newly sampled from China, Dominican Republic, Germany, Italy, South Africa, Thailand, the United Kingdom and United States of America and deposited in HMAS, PREM, HKAS (Herbarium of Cryptogams, Kunming Institute of Botany, Chinese Academy of Sciences) and MFLU. Twelve out of the 16 recognised species, plus two recently described species, as well as two putative new species and a new combination were represented in this study. Morphological characters were studied from field notes, colour images of the material and complemented with literature data. Colour names and codes are from Kornerup and Wanscher (1978). Microscopic character observations were conducted under a light microscope using thin handmade sections rehydrated in 5% aqueous potassium hydroxide (KOH) (w/v). Melzer’s reagent was used to test the amyloidity of basidiospores and cresyl blue was used to study the metachromatic reaction (Largent et al. 1977). In the descriptions of basidiospores, the abbreviation [*n*/*m*/*p*] indicates *n* basidiospores measured from *m* basidiocarps of *p* collections; (a)b–c(d) stands for the dimensions of the basidiospores, with b–c containing a minimum of 90 % of the measured values and (a) and (d) extreme values. Q is used to mean “length/width ratio” of a basidiospore and Qav represents average of Q of all basidiospores studied.

### DNA extraction, primers, PCR and sequencing

A small piece of dried basidiocarp was excised from a specimen and ground in an Eppendorf tube. Genomic DNA was isolated using the CTAB method (Doyle and Doyle 1987). Optimal dilutions of the DNAs were used to amplify the following regions: internal transcribed spacer (ITS), the large subunit nuclear ribosomal DNA (nrLSU), the second largest subunit of RNA polymerase II (*rpb2*) and the translation elongation factor 1-α gene (*tef1*). PCR amplifications used the previously described primers: ITS1F/ITS4 for ITS, LR0R/LR5 for nrLSU (Gardes and Bruns 1993; White et al. 1990), bRPB2-6F /bRPB2-7.1R for *rpb2* (Matheny 2005) and 983F/1567R for *tef1* (Rehner and Buckley 2005). PCR conditions were as recommended by the Taq polymerase manufacturer (Bioteke, Beijing, China), using an ABI 2720 Thermal Cycler (Applied Biosystems, Foster City, CA, USA). PCR products were cleaned and sequenced by Sangon Biotech (Shanghai) Co. Ltd. (Shanghai, China) and Kunming Shuoqing Biotech Ltd (Kunming, China).

### Phylogenetic analyses

**Figure 1.**
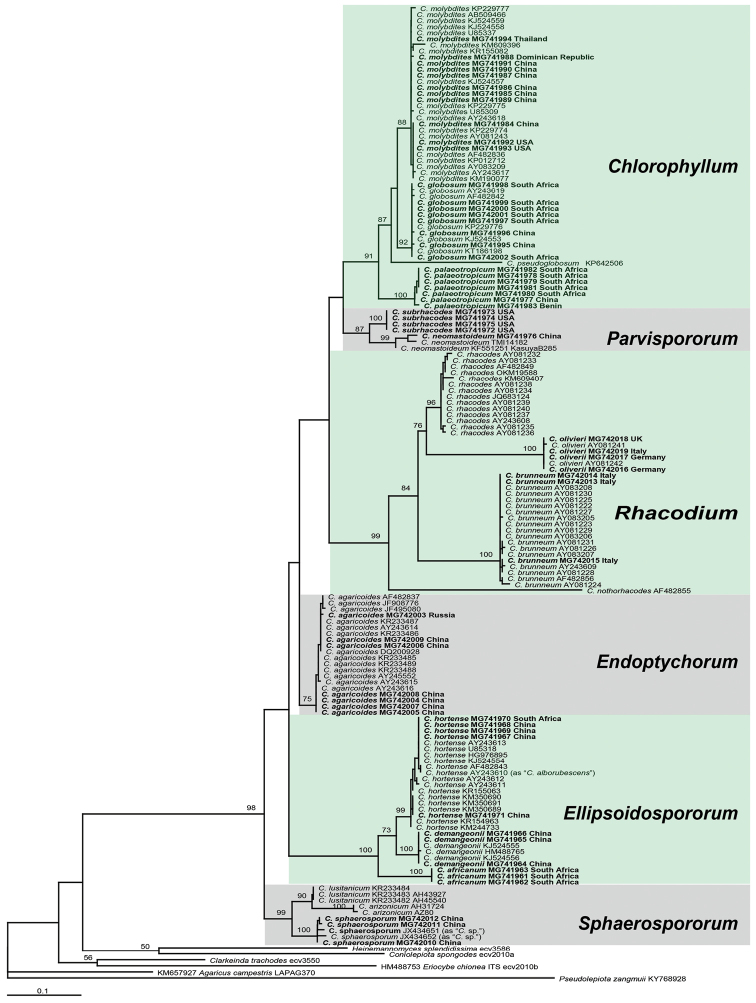
ML tree inferred from ITS data. Bootstrap values >50 % are indicated at internodes. Names of new taxa are in bold.

In this study, 144 sequences were produced using standard methods and deposited in GenBank (MG741961–MG742106), viz., 59 ITS, 29 nrLSU, 28 *rpb2* and 28 *tef1* (Figure 1 and Table 1) sequences. To obtain an estimate of *Chlorophyllum* genetic diversity, 96 ITS sequences were also retrieved from GenBank and included in the phylogenetic analyses (GenBank nos. included in Figure 1).

The ITS data set included 155 *Chlorophyllum* sequences. From these, a subset of 29 collections was chosen to represent the full range of phylogenetic diversity sampled for a four-locus dataset comprising portions of the ITS, nrLSU, *rpb2* and *tef1* (Table 1). To test the monophyly of *Chlorophyllum* within *Agaricaceae*, ML analysis of the *rpb2* dataset, which has much fewer ambiguous aligned sections in the matrix compared to the ITS dataset, was conducted with representative genera of the *Agaricaceae* (Ge and Yang 2017; Ge et al. 2015; Vellinga et al. 2011). As *Chlorophyllum* is confirmed as a monophyletic group close to *Agaricus* L. and allied genera in the present study (Suppl. material 1) and recent studies (Ge and Yang 2017; Ge et al. 2015; Vellinga et al. 2011), representative species of these genera were included as outgroups for rooting purposes for analyses; these are *Agaricus campestris* L., *Clarkeinda trachodes* (Berk.) Singer, *Coniolepiota spongodes* (Berk. & Broome) Vellinga, *Eriocybe chionea* Vellinga, *Heinemannomyces splendidissimus* Watling and *Pseudolepiota zangmui* Z.W. Ge.

Sequences were aligned using MAFFT 6.8 (Katoh et al. 2009) and further optimised visually. The best-fit evolutionary model for each dataset was determined using MRMODELTEST (Nylander 2004). Maximum Likelihood (ML) analyses were conducted in RAxML 7.2.6-WIN with default settings using a GTRGAMMA model (Stamatakis et al. 2007). Clade robustness was assessed using a bootstrap analysis with 1000 replicates (Felsenstein 1985).

**Table 1.** Taxa, vouchers, geographic origin, and GenBank accession numbers of DNA sequences of *Chlorophyllum* and outgroups used in this study. New sequences generated by this work are in bold.

Taxon	Collection	Origin	nrITS	nrLSU	*rpb2*	*tef1*
*Chlorophyllum africanum*	PREM 62140	South Africa	**MG741961**	**MG742041**	**MG742070**	**MG742098**
*C. africanum*	PREM 62141	South Africa	**MG741963**	**MG742042**	**MG742071**	**MG742099**
*C. agaricoides*	HKAS 101312	Russia	**MG742003**	**MG742020**	**MG742050**	**MG742078**
*C. agaricoides*	HMAS 71678	China: Neimenggu	**MG742004**	**MG742021**	**MG742051**	**MG742079**
*C. arizonicum*	AH31724	Mexico	KR233490	KR233499	N/A	N/A
*C. arizonicum*	Trappe 11481 (AZ80)	USA	HQ020416	HQ020419	N/A	N/A
*C. brunneum*	HKAS 101315	Italy	**MG742013**	**MG742022**	**MG742052**	**MG742080**
*C. brunneum*			AY083206	AF482886	HM488804	HM488886
*C. demangei*	Z. W. Ge 3112	China: Yunnan	**MG741965**	**MG742027**	**MG742056**	**MG742084**
*C. demangei*	Z. W. Ge 3574	China: Yunnan	**MG741964**	**MG742025**	**MG742055**	**MG742083**
*C. globosum*	Z. W. Ge 2006-1	China: Yunnan	**MG741995**	**MG742023**	N/A	N/A
*C. globosum*	PREM 62147	South Africa	**MG742002**	**MG742024**	**MG742053**	**MG742081**
*C. hortense*	HKAS 101317	China: Hainan	**MG741967**	**MG742026**	**MG742054**	**MG742082**
*C. hortense*	Z. W. Ge 3115	China: Yunnan	**MG741968**	**MG742028**	**MG742057**	**MG742085**
*C. hortense*	HKAS 90470	China: Yunnan	**MG741971**	**MG742029**	**MG742058**	**MG742086**
*C. lusitanicum*	AH45540	Spain	KR233482	KR233491	N/A	N/A
*C. lusitanicum*	AH43927	Spain	KR233483	KR233492	N/A	N/A
*C. molybdites*	HKAS 45051	China: Hunan	**MG741985**	**MG742030**	**MG742059**	**MG742087**
*C. molybdites*	Z. W. Ge 3381	USA: Florida	**MG741993**	**MG742034**	**MG742063**	**MG742091**
*C. molybdites*	Z. W. Ge 3146	China: Yunnan	**MG741987**	**MG742031**	**MG742060**	**MG742088**
*C. molybdites*	HKAS 101322	Italy	**MG741988**	**MG742032**	**MG742061**	**MG742089**
*C. molybdites*	Z. W. Ge 3377	USA: Florida	**MG741992**	**MG742033**	**MG742062**	**MG742090**
*C. neomastoideum*	HKAS 83208	China: Zhejiang	**MG741976**	**MG742035**	**MG742064**	**MG742092**
*C. olivieri*	HKAS 31587	Germany: Marburg	**MG742016**	**MG742036**	**MG742065**	**MG742093**
*C. olivieri*	HKAS 53466	Germany: Marburg	**MG742017**	**MG742037**	**MG742066**	**MG742094**
*C. palaeotropicum*	PREM 62142	South Africa	**MG741978**	**MG742038**	**MG742067**	**MG742095**
*C. palaeotropicum*	PREM 62145	South Africa	**MG741982**	**MG742039**	**MG742068**	**MG742096**
*C. palaeotropicum*	HKAS 93747	Benin: Okpara	**MG741983**	**MG742040**	**MG742069**	**MG742097**
*C. pseudoglobosum*	AM155	India: West Bengal	KP642506	KR080484	N/A	N/A
*C. rhacodes*			AF482849	AY176345	N/A	HM488885
*C. rhacodes*			U85312	U85277	HM488803	KC884736
*C. sphaerosporum*	HMAS 66153	China: Neimenggu	**MG742011**	**MG742043**	**MG742072**	**MG742100**
*C. sphaerosporum*	HMAS 71683	China: Neimenggu	**MG742012**	**MG742044**	**MG742073**	**MG742101**
*C. subrhacodes*	Z. W. Ge 3411	USA: Florida	**MG741975**	**MG742045**	**MG742074**	**MG742102**
*C. subrhacodes*	Z. W. Ge 3232	USA: Florida	**MG741973**	**MG742046**	**MG742075**	**MG742103**
*C. subrhacodes*	Z. W. Ge 3385	USA: Florida	**MG741972**	**MG742048**	**MG742077**	**MG742105**
*C. subrhacodes*	Z. W. Ge 3242	USA: Florida	**MG741974**	**MG742047**	**MG742076**	**MG742104**
**Outgroups**						
*Agaricus campestris*			KM657927	KR006607	KT951556	KR006636
*Clarkeinda trachodes*			HM488751	KY418837	HM488802	N/A
*Coniolepiota spongodes*	png012	Thailand	HM488756	HM488774	HM488796	HM488883
*Eriocybe chionea*			HM488753	HM488772	HM488800	N/A
*Heinemannomyces splendidissimus*			HM488760	HM488769	HM488793	KT951657
*Pseudolepiota zangmui*	Z. W. Ge 2175	China: Yunnan	KY768928	**MG742049**	KY768929	**MG742106**

The ITS-nrLSU, *rpb2* and *tef1* datasets were analysed separately before concatenation. As no significant (bootstrap support above 70 %) incongruence was detected, the resulting three alignments (ITS-nrLSU, *rpb2* and *tef1*) were combined for further multigene analyses. Unavailable sequences of loci from a few species were treated as missing data in the phylogenetic analyses. Final alignments have been deposited in TREEBASE (http://www.treebase.org) with accession number (S22068).

## Results

The ITS alignment contained 787 sites, all of which were included in the analyses. The ML tree is shown in Figure 1 with the final ML optimisation likelihood at -5625.939348. According to the ML tree, 17 phylogenetic species were recovered. The new species were nested within *Ellipsoidospororum* clade (*C. africanum*) and *Chlorophyllum* clade (*C. palaeotropicum*).

**Figure 2.**
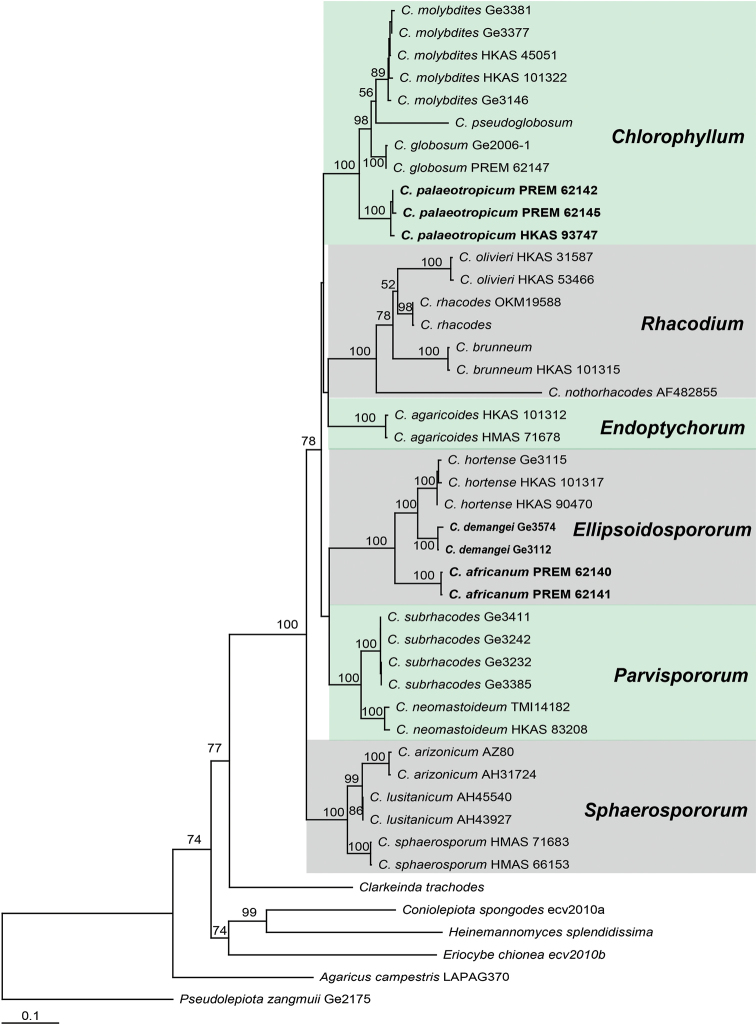
ML tree inferred from the combined alignment based on ITS, nrLSU, *rpb2* and *tef1*. Bootstrap values >50 % are indicated at internodes. Names of new taxa are shown in bold.

The combined data set included subsamples of the 17 species recovered in the ITS tree. This alignment contained 2896 nucleotide sites (including gaps), consisting of 785, 851, 699 and 561 sites (including gaps) for ITS, nrLSU, *rpb2* and *tef1*, respectively. The analyses identified six distinct well-supported clades within *Chlorophyllum*, each representing one to four species (Figure 2). These clades are: *Chlorophyllum* clade, *Ellipsoidospororum* clade, *Endoptychorum* clade, *Rhacodium* clade, *Parvispororum* clade and *Sphaerospororum* clade. Each of these clades received 100 percent maximum boot strap (ML-BP) support in the combined data set and strong support (≥87 % boot strap support) in the individual data sets (ITS-nrLSU, *rpb2* and *tef1* respectively). Species relationships within these six clades are largely resolved, but relationships amongst all clades were not resolved with confidence.

Emphasising both molecular data and morphological characters, a new infrageneric classification for *Chlorophyllum* and two new distinct species, *C. africanum* and *C. palaeotropicum* are proposed.

## Taxonomy

### Infrageneric classification of Chlorophyllum

The genus *Chlorophyllum* is divided into six sections: sect. Chlorophyllum, Sect. Ellipsoidospororum, sect. Rhacodium, sect. Parvispororum, sect. Endoptychorum and sect. Sphaerospororum.

#### Chlorophyllum sect. Chlorophyllum

Taxon classificationFungiAgaricalesAgaricaceae

Massee

##### Type.

*Chlorophyllum molybdites* (G. Mey.) Massee. Bull. Misc. Inf., Kew: 136. 1898.

≡ *Agaricus molybdites* G. Mey., Prim. fl. esseq.: 300. 1818.

##### Description.

Basidiocarps medium to large sized, stout, agaricoid, with obvious plate-like squamules. Basidiospores olive to greenish-white, thick-walled, ellipsoid to amygdaliform with a truncate apex, except for *C. palaeotropicum*, which produce subglobose basidiospores without germ pore. Cheilocystidia broadly clavate to sphaeropedunculate. Pileipellis is a palisade of hyphae with terminal elements clavate to subfusiform.

##### Discussion.

This section contains the type species of this genus, *C. molybdites* and also *C. globosum*, *C. pseudoglobosum*, as well as the novel taxon *C. palaeotropicum*.

#### Chlorophyllum sect. Ellipsoidospororum

Taxon classificationFungiAgaricalesAgaricaceae

Z.W. Ge sect. nov.

823853

##### Diagnosis.

Differs from other sections by the slender basidiocarps with furfuraceous squamules on the pileus, the non-pored, ellipsoid basidiospores and subcylindric to slightly fusiform cheilocystidia.

##### Type.

*Chlorophyllum hortense* (Murrill) Vellinga, Mycotaxon 83: 416. 2002.

≡ *Lepiota hortensis* Murrill, N. Amer. Fl. (New York) 10 (1): 59. 1917.

##### Description.

Basidiocarps agaricoid, small to medium sized, with furfuraceous squamules. Basidiospores ellipsoid to ovoid without germ pore. Cheilocystidia narrowly clavate to subcylindrical. Pileipellis a loose hymeniderm made up of clavate to subfusiform hyphae.

##### Discussion.

This section is represented by *C. hortense*, *C. demangei* and the new taxon *C. africanum. Chlorophyllum alborubescens* (Hongo) Vellinga, *C. humei* (Murrill) Vellinga, *C. mammillatum* (Murrill) Vellinga, *C. subfulvidiscum* (Murrill) Vellinga, *Leucoagaricus bisporus* Heinem., which were treated as synonyms of *C. hortense* (Murrill) Vellinga (Vellinga 2003; Akers and Sundberg 1997) also belong here (Figure 1).

#### Chlorophyllum sect. Endoptychorum

Taxon classificationFungiAgaricalesAgaricaceae

(Czernajew) Z.W. Ge, comb. & stat. nov.

823854

##### Basionym.

*Endoptychum* Czern., Bull. Soc. Imp. nat. Moscou 18(2, III): 146 1845.

##### Type.

*Chlorophyllum agaricoides* (Czern.) Vellinga, Mycotaxon 83: 416. 2002.

≡ *Endoptychum agaricoides* Czern., Bull. Soc. Imp. nat. Moscou 18(2, III): 148. 1845.

##### Description.

Basidiocarps secotioid, with inconspicuous squamules. Basidiospores thick-walled, without germ pore.

##### Discussion.

This section currently contains only one species (*C. agaricoides*), known from America, Asia and Europe.

#### Chlorophyllum sect. Parvispororum

Taxon classificationFungiAgaricalesAgaricaceae

Z.W. Ge sect. nov.

823859

##### Diagnosis.

Differs from other sections by the relatively smaller, porous basidiospores (less than 10 μm long) and a pileipellis composed of a palisade of hyphae with cylindrical terminal elements.

##### Type.

*Chlorophyllum subrhacodes* (Murrill) Vellinga, Mycotaxon 83: 416. 2002.

≡ *Lepiota subrhacodes* Murrill, Lloydia 6: 223. 1943.

##### Description.

Basidiocarps small to medium-sized, agaricoid, covered with large squamules contrasting in colour with the background. Basidiospores relatively small (less than 10 μm long), with germ pore, forming a truncated apex. Cheilocystidia clavate to mucronate clavate. Pileipellis a palisade of hyphae with cylindrical terminal elements. Hyphae without clamp connections.

##### Discussion.

This section contains the species from south-eastern North America (*C. subrhacodes*) and east Asia (*C. neomastoideum*) displaying an America-Asia disjunct distribution.

#### Chlorophyllum sect. Rhacodium

Taxon classificationFungiAgaricalesAgaricaceae

Z.W. Ge sect. nov.

823865

##### Diagnosis.

Differs from other sections by the stout basidiocarps with plate- like squamules on the pileus and white lamellae, basidiospores with wide germ pore and pileipellis composed of a tightly packed hymeniderm of cylindrical and flexuous, or narrowly clavate or narrowly lageniform elements.

##### Type.

*Chlorophyllum rhacodes* (Vittad.) Vellinga, Mycotaxon 83: 416. 2002.

≡ *Agaricus rhacodes* Vittad. [as ‘rachodes’], Descr. fung. mang. Italia: 158. 1835.

##### Description.

Basidiocarps medium to large sized, stout, agaricoid, with plate like squamules, basidiospores with wide germ pore, forming a truncated apex. Cheilocystidia clavate to sphaeropedunculate. Pileipellis a tightly packed hymeniderm of cylindrical and flexuous, or narrowly clavate or narrowly lageniform elements.

##### Discussion.

This section contains *C. nothorhacodes*, *C. brunneum*, *C. rhacodes*, *C. olivieri* and *C. venenatum* (Bon) C. Lange & Vellinga. There was controversy over the spelling of the species epithet ‘*rhacodes*’ (originally published as ‘*rachodes*’). The Nomenclature Committee for Fungi debated the issue for years and the General Committee made the final decision that the epithet of *Agaricus rhacodes* Vittad. (Descr. Fung. Mang.: 158. 1833) is to be so spelled, even though it was originally spelled ‘*rachodes*’, which was approved by the International Botanical Congress in Shenzhen, China (Wilson 2017).

#### Chlorophyllum sect. Sphaerospororum

Taxon classificationFungiAgaricalesAgaricaceae

Z.W. Ge sect. nov.

823860

##### Diagnosis.

Differs from other sections by the nonporous, globose to subglobose basidiospores and gasteroid basidiocarps or globose to subglobose basidiospores and a hymenodermal pileipellis made up of loosely arranged clavate to broadly clavate elements when the basidiocarps are agaricoid.

##### Type.

*Chlorophyllum sphaerosporum* Z.W. Ge & Zhu L. Yang, Mycotaxon 96: 187. 2006.

##### Description.

Basidiocarps agaricoid or gasteroid, covered with inconspicuous squamules. Basidiospores subglobose to globose without germ pore (with rounded apex). Cheilocystidia (if present) clavate to broadly clavate. Pileipellis a hymeniderm made up of loosely arranged clavate to broadly clavate elements.

##### Discussion.

This section contains the agaricoid *C. sphaerosporum* and two hypogeous taxa, *C. arizonicum* and *C. lusitanicum*. It is so far the only clade containing gasteroid species.

### Recognition of two new species and the transfer of *Lepiota demangei* from *Lepiota* to *Chlorophyllum*

#### Chlorophyllum africanum

Taxon classificationFungiAgaricalesAgaricaceae

Z.W. Ge & A. Jacobs, sp. nov .

823861

Figs 3A , 4

##### Diagnosis.

This species is distinguished from other *Chlorophyllum* species by relatively small basidiocarps with yellow grey to grey orange (5B4) furfuraceous squamules, the squamules composed of a hymenidermal layer made up of greyish-yellow to dull yellow, narrowly clavate to clavate elements, the hyaline ellipsoid basidiospores without a germ pore and the hyaline, cylindric to slightly fusiform cheilocystidia.

##### Type.

SOUTH AFRICA. 2229 BB Beit Bridge, Farm Matolege 133 MS (−22°14.91'S, 29°47.29'E), alt. ca. 560 m, growing in disturbed area with large volume of animal droppings, 9 February 2014, Van Der Walt, R 885 (holotype: PREM 62143!; isotype: HKAS!). ITS barcoding sequence: MG741962.

**Figure 3.**
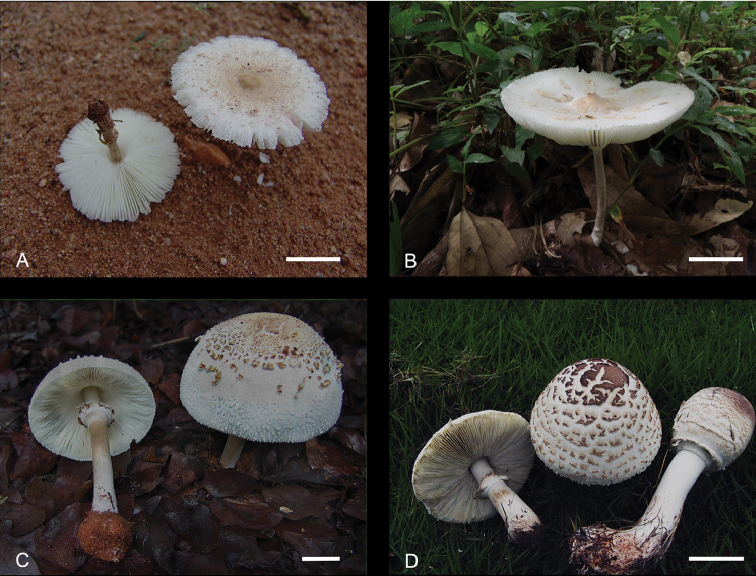
Basidiocarps of representative species of *Chlorophyllum*. **A** *Chlorophyllum africanum* (PREM 62141) **B** *Chlorophyllum demangei* (HKAS 89157) **C** *Chlorophyllum globosum* (PREM 62152) **D** *Chlorophyllum palaeotropicum* (HKAS 60195).

**Figure 4.**
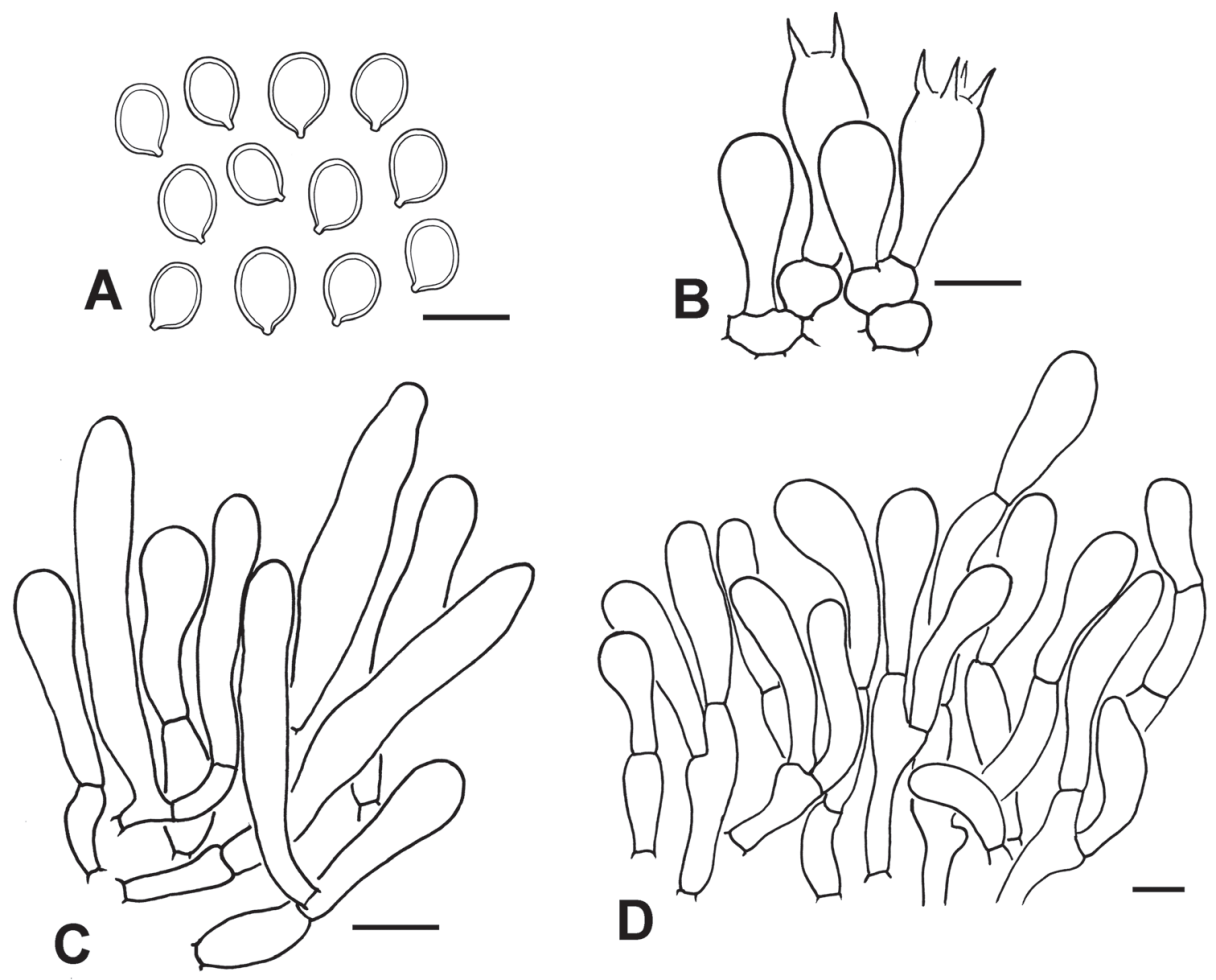
Micro-morphological features of *C. africanum* (PREM 62143, type). **A** Basidiospores **B** Basidia **C** Cheilocystidia **D** Elements of squamules on pileus. Scale bars: 10 μm (**A, D**); 20 μm (**B, C**).

##### Description.

Pileus 30–50 mm broad, hemispherical to convex when young, expanding to broadly convex or applanate with age, sometimes with a prominent umbo; margin sulcate striate; surface covered with thin, yellow grey (4B2–4B3), orange grey (6B2) to grey orange (5B4) furfuraceous squamules, these remaining intact on the disc but elsewhere diffracted-scaly with expansion and receding from pileus margin. Lamellae free and remote from stipe, white to off white, crowded, narrow, up to 6 mm deep, with 1–2 series of lamellulae; edges entire, white. Stipe 35–60 × 3–6 mm, subcylindric, slightly enlarged at base, glabrous, white to light brown, hollow, nearly stuffed, with a simple annulus about 10–15 mm from top of the stipe. Context white, 1–2 mm thick in pileus, discolouring brown to red where bruised or handled, with strong mushroom odour, taste mild. Spore print white to cream.

Basidiospores [100,5,2] (7.5)8.0–9.0 × (5.5) 6.0–6.5(7.0) μm (mean 8.2 ± 0.4 × 6.2 ± 0.3 μm), Q = (1.2)1.3–1.4 (1.5), Qav = 1.3 ± 0.05, ellipsoid, occasionally ovoid in side view or in frontal view, with rounded apex, smooth, hyaline, congophilous, dextrinoid, with one guttule in most cases, without germ pore, slightly thick walled, becoming purplish-red (14A6–14A7) in cresyl blue. Basidia 29–33 × 10.0–11.0 µm, clavate, hyaline, 4-spored, rarely 2-spored. Cheilocystidia 28–50 × 6.0–10.0 µm, cylindric to slightly fusiform, hyaline. Pleurocystidia not observed. Lamella trama regular to slightly interwoven, made up of subcylindrical hyaline hyphae, 7.0–12.0 μm diam. Pileipellis a hymenidermal layer made up of greyish-yellow (1B4, 2B3) to dull yellow (3B3), clavate elements of 21–50 × 9.0–14.0(16.0) µm, slightly thick walled, with greyish-yellow vacuolar pigments; wall greyish-yellow; terminal elements mostly narrowly clavate. Clamp connections not observed.

##### Distribution.

So far, only known from South Africa.

##### Ecology.

Saprotrophic, solitary to scattered, terrestrial.

##### Etymologyл

(L.) in reference to Africa where it is collected.

##### Additional specimens examined.

SOUTH AFRICA. 2229 BB Beit Bridge, Farm Matolege 133MS, −22°14.66'S, 29°46.75'E, 574 m, on soil. 10 January 2014, Van Der Walt, R787 (PREM 62140). 2229 BD Kamkusi, Farm Ludwigslust 163 MS (Farm Yard), −22°16.64'S, 29°48.22'E, alt. ca. 610 m, 9 March 2014, Van Der Walt, R935 (PREM 62141). Scattered in sandy soil of semi-shade to full sun, cleared area.

##### Discussion.

*Chlorophyllum africanum* is morphologically very similar to *C. bharatense* Sathe & S.M. Kulk. Both species have a small-sized convex to applanate pileus covered with pale olivaceous brown squamules, clavate cheilocystidia and broadly ellipsoid basidiospores. However, *C. bharatense* differs from *C. africanum* by the umbonate pileus, lamellae that become reddish- brown on drying, basidiospores with an indistinct or absent germpore and squamules composed of a trichodermal layer (Sathe et al. 1981 (‘1980’)).

*Chlorophyllum africanum* is also similar to *C. hortense* on account of the small-sized basidiocarps, ellipsoid basidiospores and subcylindrical cheilocystidia. However, *C. hortense* differs from *C. africanum* by 2-spored basidia and the whitish context of the stipe becoming reddish where bruised (Akers and Sundberg 1997; Vellinga 2003b).

*Chlorophyllum demangei* (see below) is characterised by the frequent and obviously umbonate pileus and large basidiospores measuring (7.5) 8.0–10.5 (12.5) × (5.0) 5.5–7.0 (7.5) µm. Molecular phylogenetic results clearly support the recognition of the two as separate species.

*Leucocoprinus zeylanicus* (Berk.) Boedijn, described from Sri Lanka, is also similar to *C. africanum* due to the small-sized pileus with a distinct umbo, the subcylindric cheilocystidia and the short ellipsoid basidiospores (Pegler 1986). However, the finely radially silky-striate pileus of *Lc. zeylanicus* beset with sparse, minute, blackish-brown repent squamules and the basidiospores with a small germ pore (Pegler 1986), differentiate this species from *C. africanum*.

*Lepiota zeyheri* (Berk.) Sacc., a species also found in South Africa, is somewhat similar to *C. africanum* on account of the whitish pileus with a clay brown umbo that is elsewhere covered with cream or brown squamules and the ellipsoid basidiospores. However, *L. zeyheri* has much larger basidiocarps measuring 10–22 cm or larger, a pale pink spore deposit, larger broadly ellipsoid basidiospores (15.0–17.0 × 10.0–12.0 µm) with a germ pore and clavate cheilocystidia (Pearson 1950).

#### Chlorophyllum palaeotropicum

Taxon classificationFungiAgaricalesAgaricaceae

Z.W. Ge & A. Jacobs sp. nov.

823862

Figs 3D , 5

##### Diagnosis.

This species is distinguished from other *Chlorophyllum* species by medium-sized basidiocarps with distinct brownish squamules composed of a trichodermal layer of subcylindrical brownish hyphae and slightly enlarged terminal elements, the greenish subglobose basidiospores without a germ pore and the clavate to narrowly clavate cheilocystidia with brownish to fuscous brown vacuolar pigments.

##### Type.

SOUTH AFRICA. 2229 BD Kamkusi, Farm Ludwigslust 163MS (−22°15.39'S, 29°47.48'E), alt. 582 m, near open area along dirt road, growing in loam soil, compost-rich – mopane (*Colophospermum* mopane) leaf layer, 30 November 2013, Van Der Walt, R 715 (Holotype: PREM 62142!; isotype: HKAS!). ITS barcoding sequence: MG741978.

**Figure 5.**
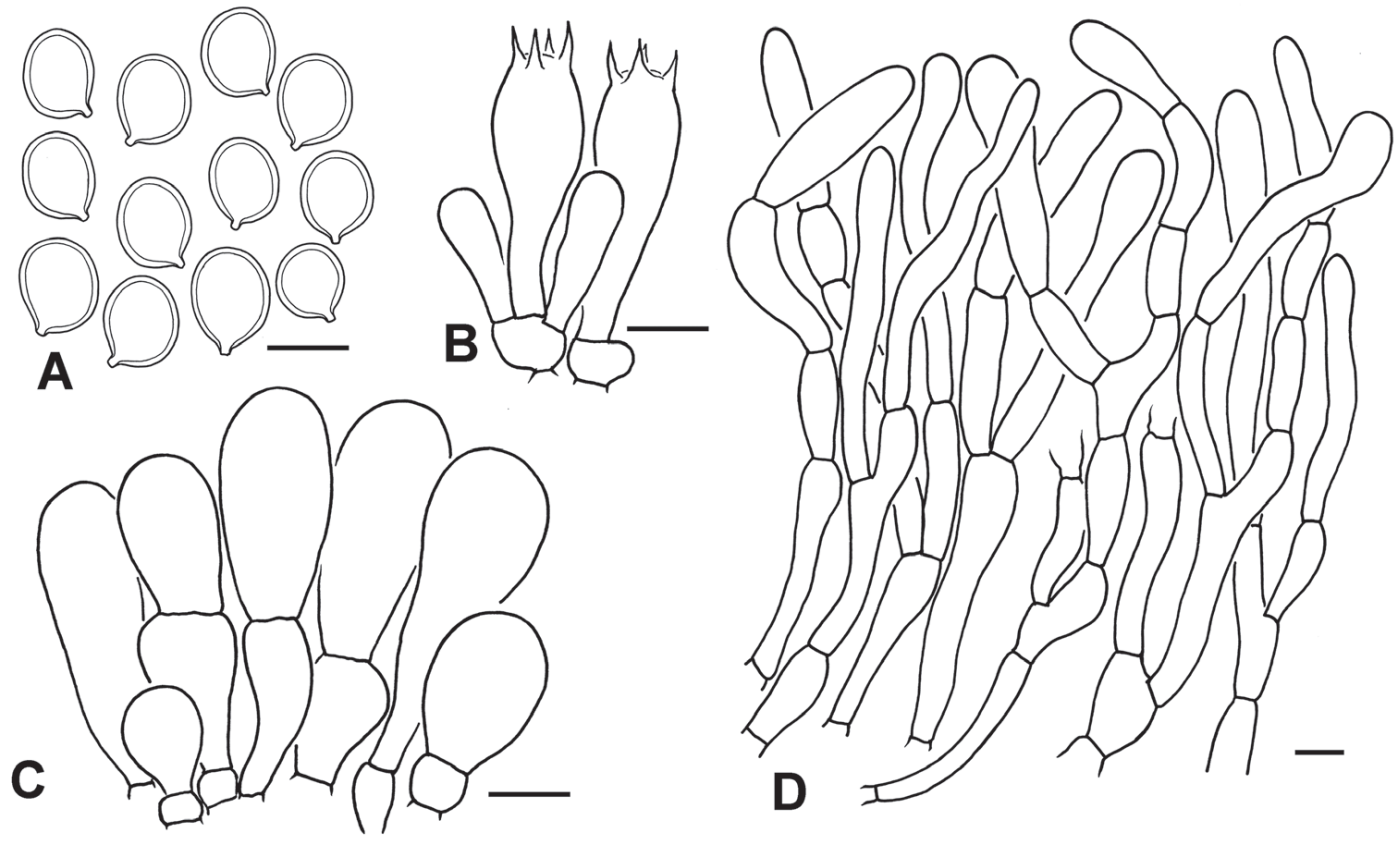
Micro-morphological features of *C. palaeotropicum* (PREM 62142, type). **A** Basidiospores **B** Basidia **C** Cheilocystidia **D** Elements of squamules on pileus. Scale bars: 10 μm (**A, D**); 20 μm (**B, C**).

##### Description.

Pileus 50–100 mm broad, hemispherical to convex at first, expanding to convex to broadly convex with age; surface covered with fibrillose, tufted, reddish-white (7A2) to brownish-orange (6C3) squamules at the margin and brownish-grey (6C2), orange grey (6B2), to greyish-brown (7D3) plate-like squamules at the centre. Lamellae free and remote from stipe; white to off-white when young, whitish to greenish-white (26A2) when mature, crowded, 6–11 mm deep, with 1–2 series of lamellulae. Stipe 16–90 × 3.5–8 mm, subcylindrical, with slightly enlarged base, straight or curved, white; hollow, nearly stuffed, with an annulus at the middle part of the stipe. Context white, 4–6 mm thick in pileus, white in pileus and stipe, discolouring pastel pink (7A4) when drying, with a distinct mushroom smell, taste mild. Spore print greyish-green (30B3–30B4).

Basidiospores [100,5,3] (8.0)8.5–11.0(12.0) × (6.0)7.0–9.0(10.0) μm (mean 9.8 ± 0.9 × 8.0 ± 0.8 μm), Q = 1.0–1.4, Qav = 1.2 ± 0.05, ellipsoid, oblong in side view or in frontal view, with rounded apex, smooth, hyaline when young, greenish-white (27A2), olive to brownish (in KOH) when mature, congophilous, dextrinoid, without germ pore, slightly thick-walled; mature basidiospores staining purplish-red (14A6–14A7) in cresyl blue; immature basidiospores staining bluish-violet (18B7) in cresyl blue. Basidia 29–33 × 10.0–12.0(15.0) µm, clavate, hyaline, 4-spored. Cheilocystidia (13)20–55(63) × 10.0–15.0(20.0) µm, clavate, rarely broadly clavate or narrowly clavate, brownish to fuscous brown, sometimes septate. Pleurocystidia absent. Lamella trama slightly interwoven, made up of subcylindrical hyaline hyphae, 8–14 μm diam. Pileipellis a trichoderm made up of filamentous or cylindrical hyphae, slightly interwoven, interspersed with brown to tea brown hyphae, 8–14 µm in diam., thick-walled, with brownish vacuolar pigments; wall brownish-yellow; terminal elements mostly slightly enlarged to narrowly clavate, rarely cylindrical. Clamp connections present on basal septa of young basidia and tissue of annulus, but not common.

##### Distribution.

Known from Benin and South Africa in Africa and from China in Asia.

##### Ecology.

Saprotrophic, solitary to scattered, terrestrial.

##### Etymology.

(L.) with reference to distribution of this species in the Old World tropics.

##### Additional specimens examined.

BENIN. Okpara: countryside of North-eastern Parakou, 15 km from Parakou, alt. ca 330 m, 18 June, 2015, G. Wu 1370 (HKAS 93747). CHINA. Hainan Province: Sanya, Yalongwan, on man-made lawn near seaside, 29 June 2010, Z.W. Ge 2519 (HKAS 60195). SOUTH AFRICA. 2229 BB Beit Bridge, Farm Wimpsh 139 MS, 12 February 2014, Van Der Walt, R891 (PREM 62144), growing in cleared area, near water hole, among *Bulbostylis hispidula*; 2229 BD Kamkusi, Farm Ludwigslust 163 MS (farm yard), −22°16.64'S, 29°48.22'E, alt. 606 m, growing in loam soil in cleared area, in semi-shade to full sun, 9 March 2014, Van Der Walt, R 938 (PREM 62145); 2229 BD Kamkusi, Farm Ludwigslust 163MS (−22°16.64'S, 29°48.22'E), alt. ca. 610 m, growing in loam soil in cleared area, 14 February 2014, Van Der Walt, R 905 (PREM 62146).

##### Discussion.

*Chlorophyllum palaeotropicum* is very similar morphologically to *C. shimogaense* Sathe & S.M. Kulk. Both species have medium-sized, hemispherical to convex pilei covered with reddish-white to brownish-orange squamules composed of a trichodermal layer. Both species also possess clavate cheilocystidia and subglobose basidiospores. However, *C. shimogaense* possesses an umbonate pileus, basidiospores with an indistinct or absent germ pore, much smaller basidia (13–24 × 6–8.5 μm) and no clamp connections (Sathe et al. 1981 (‘1980’)).

*Chlorophyllum palaeotropicum* is also similar to *C. molybdites*, *C. globosum* and *C. pseudoglobosum* in general appearance due to the brownish to reddish discolourations where bruised, but *C. palaeotropicum* differs from these three species in having subglobose to globose basidiospores without a germ pore (apex rounded), while *C. molybdites*, *C. globosum* and *C. pseudoglobosum* have amygdaliform basidiospores with large germ pores (apex truncate).

*Chlorophyllum palaeotropicum* also resembles *C. sphaerosporum* on account of the basidiocarps bearing similar subglobose basidiospores without a germ pore. However, the squamules of *C. sphaerosporum* are made up of a hymenidermal layer composed of clavate to broadly clavate terminal elements. Furthermore, the context of *C. sphaerosporum* does not change colour when bruised. So far, *C. sphaerosporum* has only been recorded from temperate regions in northern China. These two species also belong to two different sections (Figure 1).

*Chlorophyllum palaeotropicum* is somewhat similar to *L. zeyheri* on account of the overall appearance, the broadly ellipsoid basidiospores and clavate cheilocystidia. However, *L. zeyheri* has much larger basidiocarps measuring 10–22 cm or larger and pale pink spore prints (Pearson 1950). Furthermore, *L. zeyheri* has larger basidiospores (15.0–17.0 × 10.0–12.0 µm) with a germ pore (Pearson 1950).

#### Chlorophyllum demangei

Taxon classificationFungiAgaricalesAgaricaceae

(Pat.) Z.W. Ge & Zhu L. Yang comb. nov.

823863

##### Basionym.

*Lepiota demangei* Pat., Bull. trimest. Soc. mycol. Fr. 23(2): 78. 1907.

##### Type.

VIETNAM. Hanoi: Tonkin, M. Demange 236 (Herb. Patouillard, FH 4244–holotype!).

##### Description.

Pileus small to medium-sized, 2.5–8.5 cm in diam. (Figure 3B), umbonate, white to cream coloured, covered with concentrically arranged, ochraceous to yellowish-brown squamules; margin finely striate. Lamellae free, white to cream-coloured, 5–7 mm in height. Stipe 5–6 × 0.2–0.5 cm, whitish, becoming yellowish to brownish on bruising, slender, annulus 1–1.5 cm below apex of the stipe, persistent. Context of pileus and stipe white, becoming reddish, pinkish or orange red when cut, thin in pileus. Spore print white.

Basidiospores [45/2/1] (7.5) 8.0–10.0 (12.5) × (5.0) 5.5–7.0 (7.5) μm, 8.7 ± 0.4 × 6.3 ± 0.3 μm, Q= (1.3)1.4–1.7 (1.8), Qav =1.5 ± 0.09; ellipsoid, hyaline, strongly dextrinoid, slightly thick-walled, apex lacking germ pore but somewhat thinner than other areas. Basidia 25–30 × 7–9 μm, clavate, 4-spored. Squamules on pileus (pileus disc of the smaller slice of the holotype) composed of clavate to narrowly clavate cells, 45–66 × 11–15 μm, hyaline to very pale brownish in KOH. Clamp connections not observed.

##### Distribution.

Known from China and Vietnam in Asia.

##### Ecology.

Saprotrophic, solitary to scattered, terrestrial.

##### Additional specimens examined.

CHINA. Yunnan Province: Xishuanbangna Prefecture, Mengla County, Mengyuan, alt. ca. 770 m, July 2, 2014, Z.W. Ge 3574 (HKAS 84412); same locality, K. Zhao 494 (HKAS 89157); Honghe Prefecture, Gejiu City, Manghao town, September 25, 2011, Z.W. Ge 3112 (HKAS 70616).

##### Discussion.

The distinctive characters of *Chlorophyllum demangei* are the discolouration of basidiocarps when bruised, the ellipsoid basidiospores without a germ pore and the pileal squamules composed of clavate to narrowly clavate elements. From the examination of specimens newly collected from southern Yunnan in China, not far away from the locality where the type of *Lepiota demangei* was collected, the distinctive characters were found that fit the description of *Lepiota demangei* (Yang 2000) very well. Thus, *Lepiota demangei* is transferred from *Lepiota* to *Chlorophyllum*.

#### Chlorophyllum globosum

Taxon classificationFungiAgaricalesAgaricaceae

(Mossebo) Vellinga

##### Type.

CAMEROON. Yaoundé, alt. 780 m, growing on humus in shade under tree, 1 November, 1996, D. C. Mossebo, D. C. Mossebo 98-1 kept in the Herbarium of University of Yaoundé I (*non vide*).

##### Description.

Basidiocarps medium to large-sized (Figure 3C). Pileus 5.0–20.0 cm broad, ovoid to subglobose when young, expanding to parabolic, convex to broadly convex with age; margin inflexed, with short, fine striations; surface covered with yellowish-white (3A2) to yellowish-grey (4A2), greyish-yellow (4B3–4B4), brownish-orange (6C6) to greyish-brown (5D3) squamules. The squamules remain intact at disc, but elsewhere diffract with expansion and recede from pileus margin, displaying the white to yellowish-white (2A2, 3A2, 4A2) felted or fibrillose background which turned pastel red to red (9A4–6) when touched. Lamellae free and remote from stipe with obvious gutter, white to orange-white (5A1–2) when young, turning pastel red to red (9A4–6) when touched, pastel green to greyish-green (29A4, 29B4) when fully mature, crowded, ventricose and narrow near pileal margin, crowded, up to 8 mm wide, with 1–2 series of lamellulae; edge finely fimbriate, white to yellowish-grey (4A2). Stipe 8.5–28.0 × 1.0–3.1 cm, subcylindric, tapering to apex, with bulb-like, 3.0–3.4 cm wide; glabrous, white to brownish-orange (6C3–6), hollow, nearly stuffed, with an annulus about 1/3 away from the stipe apex (Figure 3C); sometimes with distant white fibrillose at apex zone, turning pastel red to red (9A4–6) when touched, with white rhizomorph connected to substrate. Context thick, white in pileus and stipe, brownish-orange (6C3–6) at apex zone, paler to middle zone and white downward base, discolouring pastel red to red (9A4–6) in both pileus and stipe context when bruised, with mushroom odour. Taste mild. Spore print yellowish-white (2A2) to pale yellow (2A3) to greyish-green (29D3–5, 29D5–6).

**Figure 6.**
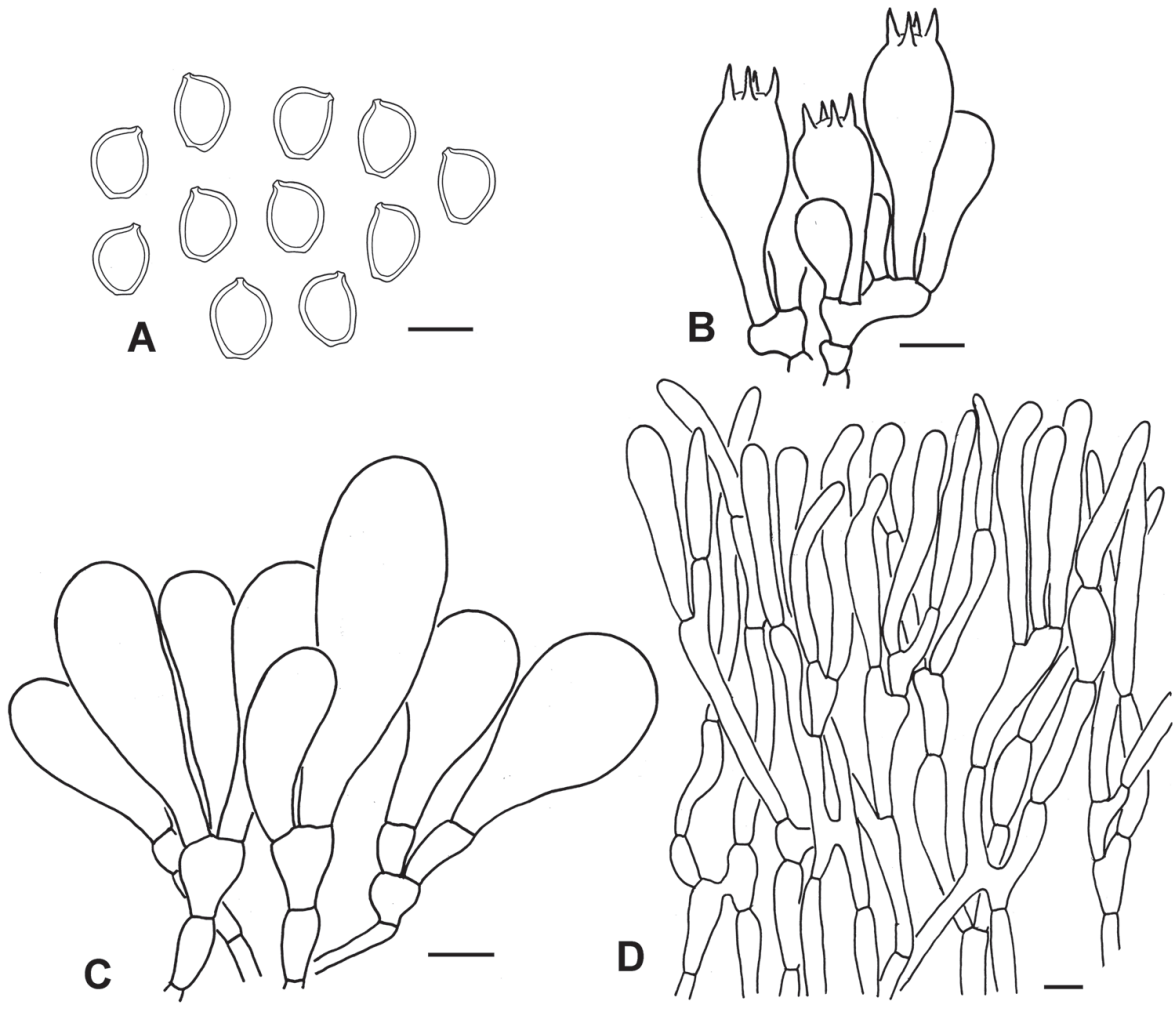
Micro-morphological features of *C. globosum* (HKAS 52741). **A** Basidiospores **B** Basidia **C** Cheilocystidia **D** Elements of squamules on pileus. Scale bars: 10 μm (**A, D**); 20 μm (**B, C**).

Basidiospores [40,2,2] (10.5)11.5–12.0 (12.5) × (8.0) 8.5–9.0 μm (mean 11.8 ± 0.4 × 8.7 ± 0.3 μm), Q = 1.3–1.4 (1.5), Qav = 1.4 ± 0.05, broadly amygdaliform in side view, ovoid in frontal view, with truncate apex, smooth, greenish-white (28A2), congophilous, dextrinoid, thick-walled (Figure 6A), becoming purplish-red (14A6–14A7) in cresyl blue. Basidia 29–38 × 12.0–14.0 µm, clavate, hyaline, 4-spored, sometimes 2-spored, rarely 1-spored. Cheilocystidia 42–65 × (15.0)18.0–29.0 µm, clavate occasionally with slightly long stalk, hyaline, sometimes with greyish-yellow vacuolar pigments. Pleurocystidia absent. Pileipellis a hymenidermal layer made up of subcylindrical hyphae (5.0–11.0 µm in diam.), slightly thick walled, with dull yellow (3B3) vacuolar pigments; terminal elements with rounded or attenuate apex, mostly narrowly clavate. *Clamp connections* not observed.

##### Distribution.

Known from Cameroon, Nigeria and South Africa in Africa and from China, India and Thailand in Asia.

##### Ecology.

Saprotrophic, solitary to scattered, terrestrial.

##### Specimens examined.

CHINA. Yunnan Province: between Yuanmou and Yongren, 28 June 2006, Z.W. Ge 2006-1 (HKAS 52741). SOUTH AFRICA. 2229 BD Kamkusi, Farm Ludwigslust 163 MS, −22°16.27'S, 29°49.02'E, alt. ca. 580 m, 12 March 2014, Van Der Walt, R 957 (PREM 62147), growing in sandy soil under Sickle bush (*Dichrostachys cinerea*) and Umbrella thorn trees (*Vachellia tortilis*, formerly *Acacia tortilis*); 2229 BD Kamkusi, Farm Ludwigslust 163 MS, alt. 584m, growing in sandy soil under Sickle bush and Umbrella thorn trees, 7 February 2014, Van Der Walt, R 869 (PREM 62148); same locality, 9 March 2014, Van Der Walt, R 936 (PREM 62149); 2229 BB Beit Bridge, Farm Matolege 133 MS, alt. ca. 580m, shady area under blue thorn (*Senegalia erubescens*, formerly *Acacia erubescens*), compost-rich, adjacent to lawn in hunting camp, 12 February 2014, Van Der Walt, R 892 (PREM 62150); 2229 BB Beit Bridge, Farm Wimpsh 139 MS, alt. ca. 604 m, loam soil, amongst grass – adjacent to seasonally waterlogged pan, 12 January 2014, Van Der Walt, R 821 (PREM 62151); same locality, 14 February 2014, Van Der Walt, R 900 (PREM 62152). THAILAND. Chiang Mai Province: Mae Taeng District, Pongduad Village, 16°06'N, 99°43'E, 780–810 m, 16 June 2010, P. Sysouphanthong, P37 (MFLU100555); Chiang Rai Province: Muang District, Ratjabhat University campus, 30 August 2012, P. Sysouphanthong, 2012-21 (MFLU121815).

##### Discussion.

*Chlorophyllum globosum* was originally described from Cameroon in the genus *Macrolepiota*. It was said to differ from *Macrolepiota odorata* “by the globose pileus and the ochraceous spore print” (Mossebo et al. 2000). In fact, the pileus does not stay globose during maturation but becomes broadly convex (Figure 3C). Based on the morphological characters such as the truncate basidiospores and its phylogenetic position, Vellinga (2002) transferred it to *Chlorophyllum*. The authors’ molecular phylogeny confirms that *C. globosum* nests in *Chlorophyllum* close to *C. molybdites*, but it differs from the latter in having a pale yellow spore print and clavate cheilocystidia. This species was first described from Africa, but its presence in several Asian countries is confirmed.

### Key to the 17 species of *Chlorophyllum* included in the present study

**Table:** 

1	Basidiocarps sequestrate (either secotioid or gasteroid), basidiospores statismosporic	**2**
–	Basidiocarps agaricoid, basidiospores ballistosporic	**4**
2	Basidiocarps secotioid, the margin of the pileus does not break free from the stipe, hymenophore (gleba) labyrinthiform to sub-lamellate	***C. agaricoides***
–	Basidiocarps gasteroid, stipe absent or rudimentary with a thick whitish mycelial cord, gleba crossed by a columella and capillitium	**3**
3	Columella not fully developed; basidiospores 7–12 µm in diam	***C. arizonicum***
–	Columella well-developed, reaching halfway up the basidiocarp; basidiospores 10–14(–15) × 10–13(–14) µm	***C. lusitanicum***
4	Basidiocarps overall white; basidiospores without germ pore, with rounded apex	**5**
–	Basidiocarps with distinct dark brown patches and squamules; basidiospores with germ pore; apex truncated	**9**
5	Basidiospores subglobose to globose; cheilocystidia clavate to broadly clavate	**6**
–	Basidiospores ellipsoid; cheilocystidia subcylindric to slightly fusiform	**7**
6	Spore print greyish-green, known from the palaeotropical regions	***C. palaeotropicum***
–	Spore print white, known from temperate region in Northern China	***C. sphaerosporum***
7	Basidia 2-spored; widely distributed in Africa, America and Asia	***C. hortense***
–	Basidia 4-spored; known from palaeotropics	**8**
8	Pileus with obvious umbo, basidiospores measuring (7.5) 8.0–10.5 (12.5) × (5.0) 5.5–7.0 (7.5), known from Southeast Asia	***C. demangei***
–	Pileus without obvious umbo, basidiospores (7.5) 8.0–9.0 × (5.5) 6.0–6.5(7.0) μm), known from South Africa	***C. africanum***
9	Basidiospores less than 10 µm long; terminal elements of pileipellis cylindrical, basidiocarps grown in bamboo forest in east Asia or under oaks of Florida in south-eastern U.S.A	**10**
–	Basidiospores longer than 10 µm; terminal elements of pileipellis clavate to narrowly clavate; basidiocarps in various habitats (meadows, pastures, lawns, greenhouse, natural forests)	**11**
10	Cheilocystidia clavate, without apical excrescences; clamp connections present at base of basidia and cheilocystidia; distributed in Asia	***C. neomastoideum***
–	Cheilocystidia clavate, some mucronate or with apical excrescences; clamp connections absent at base of basidia and cheilocystidia; distributed in North America	***C. subrhacodes***
11	Spore print green; lamellae completely greenish with age	***C. molybdites***
–	Spore print white or off-white; lamellae whitish or brownish with age, never totally green; sometimes a bluish-green shade is present near the stipe	**12**
12	Cheilocystidia sphaeropedunculate to broadly clavate, often catenate	**13**
–	Cheilocystidia narrowly clavate to clavate	**14**
13	Pileus squamules of similar colour as background, olivaceous brown to greyish-brown	***C. olivieri***
–	Pileus squamules brown (different shades) on white to cream background, which is distinctly paler than squamules	***C. rhacodes***
14	Clamp connections absent at base of basidia and cheilocystidia, cheilocystidia clavate to fusiform; annulus relatively simple	***C. nothorhacodes***
–	Clamp connections present at base of basidia and cheilocystidia, cheilocystidia narrowly clavate to fusiform; annulus relatively simple or complex	**15**
15	Basidiocarps with abruptly to marginately bulbous stipe base; annulus relatively simple, without a double crown, but with a tough brown patch on the underside	***C. brunneum***
–	Basidiocarps with widened base of stipe, but not abruptly so; annulus complex, with double crown	**16**
16	Spore print yellowish-white to pale yellow to greyish-green, basidiospores greenish-white, 8–11 × 5–6 (7) µm	***C. globosum***
–	Spore print white, basidiospores hyaline, 10–10.7 (11) × (7) 8–8.5 (9.5) µm	***C. pseudoglobosum***

## Discussion

### *Monophyly and infrageneric classification of* Chlorophyllum

In the present study, based on the extensive dataset comprising 75 % of all known species, four gene regions were used to clarify the evolutionary relationships of *Chlorophyllum*, in separate and multi-locus analyses. Based on molecular data, the genus *Chlorophyllum* is monophyletic and the genus *Clarkeinda* appeared to be the likely sister clade to *Chlorophyllum* (Figure 2). A six-section infrageneric classification of *Chlorophyllum* was proposed based on the demarked morphological characters of well-supported clades. This phylogeny also elucidated the systematic positions of previously not included taxa, such as *C. demangei*, *C. sphaerosporum* and *C. subrhacodes* (Figure 2). In addition, two new species, *C. africanum* and *C. palaeotropicum* have been added.

### Useful morphological characters in delimitation sections and species within *Chlorophyllum*

The morphological diversity within *Chlorophyllum* is mainly reflected in the general appearance of basidiocarp, colour reaction of context when bruised, the structure of pileus squamules, colour, shape and size of basidiospores and presence / absence of germ pore, shape of cheilocystidia and presence /absence of clamp connections. Based on the morphological characters chiefly used for species delimitation, morphological features in *Chlorophyllum* appear to be fast evolving and prone to shifts and no synapomorphic characteristics have been found to consistently separate the sections. This is probably due to the fact that major evolutionary radiations might have occurred in a relatively short time as it can be seen that most of the deep branches in the phylogenies are short. Nevertheless, several character combinations are phylogenetically informative thus are useful for delineating sections and species.

**1. General habitus of basidiocarps.** The sequestrate (secotioid / gasteroid) form of basidiocarps is considered to be the result of selective pressures (Bruns et al. 1989) and the loss of forcible spore discharge has been found in several predominantly agaricoid genera within the family *Agaricaceae* (Gube and Dörfelt 2012), e.g. *Agaricus* (Vellinga 2004), *Macrolepiota* (Lebel and Syme 2012) and *Lepiota* (Ge and Smith 2013; Vidal et al. 2015). The transition from agaricoid to secotioid or gasteroid is thought to be irreversible (Hibbett 2004). The majority of *Chlorophyllum* species is agaricoid and the secotioid / gasteroid habit is an apomorphy for the genus *Chlorophyllum* and consequently can be used together with other characteristics to distinguish clades. These phylogenetic analyses demonstrate that the secotioid *C. agaricoides* forms an independent clade, while the gasteroid *C. arizonicum* and *C. lusitanicum* jointly form a clade that is sister to the agaricoid *C. sphaerosporum* (Figure 2). These results suggest that the gasteroid *C. arizonicum* and *C. lusitanicum* derived from the same ancestor as *C. sphaerosporum*, while the secotioid *C. agaricoides* may have evolved independently from a different agaricoid ancestor in the genus.

**2. Colour reaction of the context when bruised.** The context of *Chlorophyllum* species shows reddening changes when exposed to the air, from light sienna, pinkish, pinkish cinnamon, reddish, dull brownish-orange to orange red (Crous et al. 2015a; Crous et al. 2015b; Ge and Yang 2006; Vellinga 2003a, 2003b). These changes are difficult to quantify and have not been uniformly recorded according to the same criteria and thus, cannot be used to distinguish sections. Nevertheless, this character can be used in combination with other characters in delimitation of species as this character varies amongst species: some species have a strong reddening reaction, some only weakly change pinkish, in others the context becomes reddish first, then changes to brown.

**3. Structure of pileus squamules.** The squamules in *Chlorophyllum* are considered to be a hymeniform layer in general, but can be further divided into three different types: i. a palisade of hyphae with terminal clavate to subfusiform elements; ii. a tightly packed hymeniderm of cylindrical and flexuous, narrowly clavate or narrowly lageniform elements; and iii. a hymeniderm of loosely arranged clavate to subfusiform hyphae. These different types of structure of squamules can be used to delimit sections in combination with other characters. For instance, the squamules of species in section Chlorophyllum are of type i, those of section Rhacodium are of type ii, while those of section Ellipsoidosporum and section Sphaerosporum are of type iii.

**4. Colour, shape and size of basidiospores and presence/absence of germ pore.** The basidiospores of *Chlorophyllum* vary from subglobose to globose without germ pore, ellipsoid without germ pore and amygdaliform to ellipsoid with large germ pore that causes the spore apex to be obviously truncated. The “ellipsoid without germ pore” shape is a conspicuous synapomorphy for the *Ellipsoidospororum* clade. Similarly, “subglobose to globose basidiospores without a germ pore” is characteristic of the Sphaerospororum clade, while all species in section Parvispororum have relatively small (less than 10 μm long) basidiospores with a truncate apex. Basidiospores can be hyaline or olive to greenish and this character can be used to separate certain clades: species within the *Chlorophyllum* clade and *Endoptychorum* clade may have olive to greenish basidiospores, while the remaining clades have hyaline basidiospores.

**5. Shape of cheilocystidia.** Shape of cheilocystidia within *Chlorophyllum* ranges from subcylindrical, slightly fusiform, narrowly clavate, clavate, mucronate clavate, broadly clavate to sphaeropedunculate. These changes are informative in recognising certain, but not all sections. For example, the cheilocystidia of species in section Ellipsoidospororum are narrowly clavate to subcylindrical, while in other sections, the cheilocystidia are clavate to sphaeropedunculate.

**6. Presence/absence clamp connections.** Amongst the species studied in the present study, most species have clamp connections with the exception of the following five species: *C. agaricoides*, *C. africanum*, *C. demangei*, *C. nothorhacodes* and *C. subrhacodes*. Since clamp connections occur in five different sections, this character is not informative at section level.

## Conclusions and future directions

This study constitutes the first multigene molecular phylogenetic analysis of the genus *Chlorophyllum*. Previous analyses included only a limited number of ITS/nrLSU sequences. This study significantly increased the molecular sampling for this group and included a wider array of taxa from a broader geographic range. Several previously unsampled species were included (i.e. *C. africanum*, *C. demangei*, *C. palaeotropicum*, *C. sphaerosporum* and *C. subrhacodes*). Based on these results, the genus *Chlorophyllum* is monophyletic and composed of six well-supported monophyletic groups that were classified as sections (Figure 2). Each section is also characterised by several morphological features. Although the relationships amongst all sections are not yet fully resolved, relationships amongst species within sections are. The majority of *Chlorophyllum* species occurs in disturbed or arid habitats in subtropical to tropical regions and many species have a wide distribution over more than one continent. The role of humans in some of these distribution patterns should be investigated.

## Supplementary Material

XML Treatment for Chlorophyllum sect. Chlorophyllum

XML Treatment for Chlorophyllum sect. Ellipsoidospororum

XML Treatment for Chlorophyllum sect. Endoptychorum

XML Treatment for Chlorophyllum sect. Parvispororum

XML Treatment for Chlorophyllum sect. Rhacodium

XML Treatment for Chlorophyllum sect. Sphaerospororum

XML Treatment for Chlorophyllum africanum

XML Treatment for Chlorophyllum palaeotropicum

XML Treatment for Chlorophyllum demangei

XML Treatment for Chlorophyllum globosum

## Supplementary materials

Supplementary material 1 Figure S1. Maximum Likelihood tree showing the monophyly of Chlorophyllum inferred from the rpb2 data set Data type: molecular data Explanation note: Bootstrap values (>50) are indicated along nodes. The clade where *Chlorophyllum* species are nested is highlighted in grey. This is an open access article distributed under the terms of the Creative Commons Attribution License (CC BY 4.0), which permits unrestricted use, distribution, and reproduction in any medium, provided the original author and source are credited.Zai-Wei Ge, Adriaana Jacobs, Else C. Vellinga, Phongeun Sysouphanthong, Retha van der Walt, Carmine Lavorato, Yi-Feng An, Zhu L. Yang
