# Supplementary figures and images for: A multi-gene phylogeny of Chlorophyllum (Agaricaceae, Basidiomycota): new species, new combination and infrageneric classification
Source: MycoKeys. 2018 Mar 20;(32):65–90. doi: 10.3897/mycokeys.32.23831 (PMC5904524; doi:10.3897/mycokeys.32.23831)

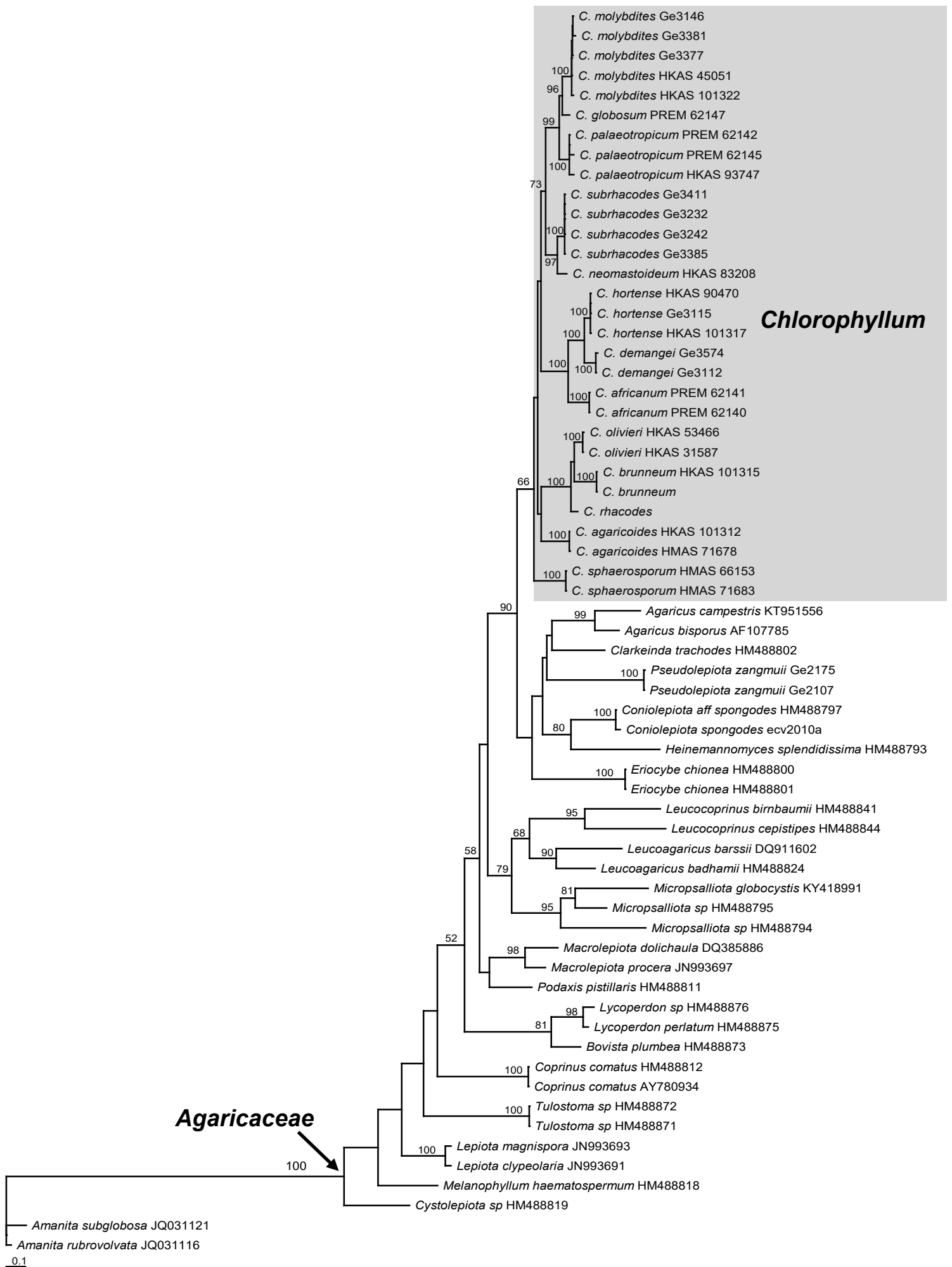

Supplement: Supplementary material 1 — Figure S1. Maximum Likelihood tree showing the monophyly of Chlorophyllum inferred from the rpb2 data set [file mycokeys-32-065-s001.pdf]
